# Visualization, Interaction and Tractometry: Dealing with Millions of Streamlines from Diffusion MRI Tractography

**DOI:** 10.3389/fninf.2017.00042

**Published:** 2017-06-26

**Authors:** Francois Rheault, Jean-Christophe Houde, Maxime Descoteaux

**Affiliations:** ^1^Sherbrooke Connectivity Imaging Lab, Computer Science Department, University of SherbrookeSherbrooke, QC, Canada; ^2^Sherbrooke Molecular Imaging Center, University of SherbrookeSherbrooke, QC, Canada; ^3^Centre de Recherche, Centre Hospitalier Universitaire de Sherbrooke (CHUS), University of SherbrookeSherbrooke, QC, Canada

**Keywords:** diffusion MRI, tractography, tractometry, connectomics, streamlines, compression, linearization, MI-Brain

## Abstract

Recently proposed tractography and connectomics approaches often require a very large number of streamlines, in the order of millions. Generating, storing and interacting with these datasets is currently quite difficult, since they require a lot of space in memory and processing time. Compression is a common approach to reduce data size. Recently such an approach has been proposed consisting in removing collinear points in the streamlines. Removing points from streamlines results in files that cannot be robustly post-processed and interacted with existing tools, which are for the most part point-based. The aim of this work is to improve visualization, interaction and tractometry algorithms to robustly handle compressed tractography datasets. Our proposed improvements are threefold: (i) An efficient loading procedure to improve visualization (reduce memory usage up to 95% for a 0.2 mm step size); (ii) interaction techniques robust to compressed tractograms; (iii) tractometry techniques robust to compressed tractograms to eliminate biased in tract-based statistics. The present work demonstrates the need of correctly handling compressed streamlines to avoid biases in future tractometry and connectomics studies.

## 1. Introduction

Diffusion magnetic resonance imaging (dMRI) tractography has greatly helped to advance the understanding of brain structural connectivity (Clayden, 2013) as it is the only non-invasive technique used to reconstruct white matter pathways (Catani and de Schotten, 2012). Tractography reconstruction algorithms produce outputs of hundreds of thousands to tens of millions of streamlines. The term streamline is used to designate the contiguous set of 3D points produced by tractography algorithms. A large quantity of streamlines generated from a single subject is called a tractogram. A typical dataset, generated using high angular resolution diffusion imaging (HARDI) tractography with a 0.5 mm step size and containing 700 k streamlines, is ~1 gigabyte (GB) on disk. When loaded in random access memory (RAM) in a visualization tool, RAM usage can go up to 7 GB depending on the datatype and internal data structures (Presseau et al., 2015).

This kind of memory usage makes these datasets barely usable on a majority of computers having only 8 or 16 GB of RAM. Another problem arises when trying to transfer these files to collaborators. The transfer can take a long time and use a lot of bandwidth. Finally, in many instances users want to manually segment streamlines in a 3D interactive visualization application. These manual virtual streamlines dissection (Catani et al., 2002) can be referred to as “interactions” with the tractogram. The main issue when interacting with such large tractograms is the large number of points, making the classical interaction techniques struggle to work responsively. Since these techniques need to quickly iterate on all the points, increasing the number of points decreases the performance and responsiveness of the application. Hence, some tools decide to partially display the tractogram, with a certain percentage of streamlines or only streamlines intersecting a certain axis (Wang et al., 2007).

Another type of approach is to use a fast streamlines clustering algorithm (Olivetti et al., 2013; Garyfallidis et al., 2016) to compute meaningful clusters at different resolutions. This level of detail approach is used in the Tractome software (Porro-Muñoz et al., 2015). When a cluster is selected, new clusters are generated with smaller thresholds. The number of geometric primitives displayed is substantially reduced while conserving relevant information for the users.

For iterative tractography reconstruction methods, the step size (distance between two consecutive 3D points) has a crucial impact on the quality of the reconstructed streamlines. Most algorithms use a uniform step size. With probabilistic tracking, which uses probabilities to determine the next stepping direction, if the step size is too small, the streamlines will look “noisy” because of the small differences in the orientation between each segments. Too big of a step size will create an overshooting problem in curved areas, resulting in an inability to reconstruct smaller U-shaped steamlines (Tournier et al., 2012). One tenth of the voxel size in diffusion space is the step size which yields reasonable streamline reconstructions in an acceptable processing time (Tournier et al., 2012; Girard et al., 2014). This was confirmed in simulations and Tractometer evaluations (Côté et al., 2013).

At a 1 mm isotropic resolution, a common white matter mask (obtained from the T1 segmentation or a thresholded FA map) contains nearly 500 k voxels. At the same resolution, a white matter and gray matter interface mask (Smith et al., 2012; Girard et al., 2014) contains approximately 200 k voxels. Each voxel can be seeded multiple time, sometimes as much as 30 seed per voxel (Hagmann et al., 2007). For clinical application, one seed per voxel is enough, but it is becoming increasingly widespread to use more than one seed per voxel. It will soon become the norm in clinical application, leading to several gigabytes per tractogram, consequently creating a storage problem for large cohorts. Novel methods such as track density imaging (TDI), tract-weighted imaging (Calamante et al., 2010, 2015) or Spherical-deconvolution Informed Filtering of Tractograms (SIFT) (Smith et al., 2015) require thousands of seeds per voxel, yielding a high number of streamlines, normally in the order of millions. Furthermore, to get the full spatial extent of specific bundles millions of streamlines need to be generated and then visualized to proceed to a virtual dissection (Gauvin et al., 2016).

In the field of connectomics, streamlines are often simplified to their 2 endpoints to represent the structural connectivity of the brain (Hagmann et al., 2008; Catani et al., 2013). In contrast, neurosurgical planning applications use the entire path, since the neurosurgeon needs to visualize white matter bundle in its entirety to be able to correctly plan the surgery (Fortin et al., 2012). Moreover, streamlines-specific statistics, called tractometry metrics (Yeatman et al., 2012; Jones and Nilsson, 2014), also use the full spatial extent of the reconstructed streamlines. Tractometry is normally computed on bundles of streamlines which were manually (Catani et al., 2002; Catani, 2005) or automatically (Guevara et al., 2011; Garyfallidis et al., 2012; O'Donnell et al., 2013; Chekir et al., 2014; Wassermann et al., 2016) segmented from a whole brain tractogram. These metrics are essential in helping neuroscientists discover relationships between the brain structure and specific diseases (Michielse et al., 2010; Sala et al., 2012). Therefore, working with datasets representing the full spatial extent of the reconstructed streamlines is necessary.

Since current tools and methods used to interact with streamlines are point-based (Soares et al., 2013), removing points can have important implications. Tools such as MRtrix (Tournier et al., 2012), FSLView (Jenkinson et al., 2012), Fibernavigator (Vaillancourt et al., 2010; Chamberland et al., 2014) or Dipy (Garyfallidis et al., 2014) rely solely on points inside or outside regions of interest (ROIs) to interact with streamlines. TrackVis (Wang et al., 2007), a frequent choice amongst neuroscientists when segmenting streamlines into anatomical bundles, uses 2 different methods to select streamlines. One is point-based (for spherical ROIs) and the other (hand drawn ROIs) uses the full path of the streamlines and does not rely solely on points. This inconsistent way to segment streamlines can have important consequences if the step size is not uniform. Without any compression, the current methods are normally able to correctly segment the vast majority of streamlines, due to the points density and the assumption of a uniform step size along streamlines. Such tractograms can be created when using global tracking or when compressing streamlines before post-processing. The reproducibility of the results and the statistics computed from streamlines are very important for the assessment of the findings (Ciccarelli et al., 2003).

**Figure 1:**
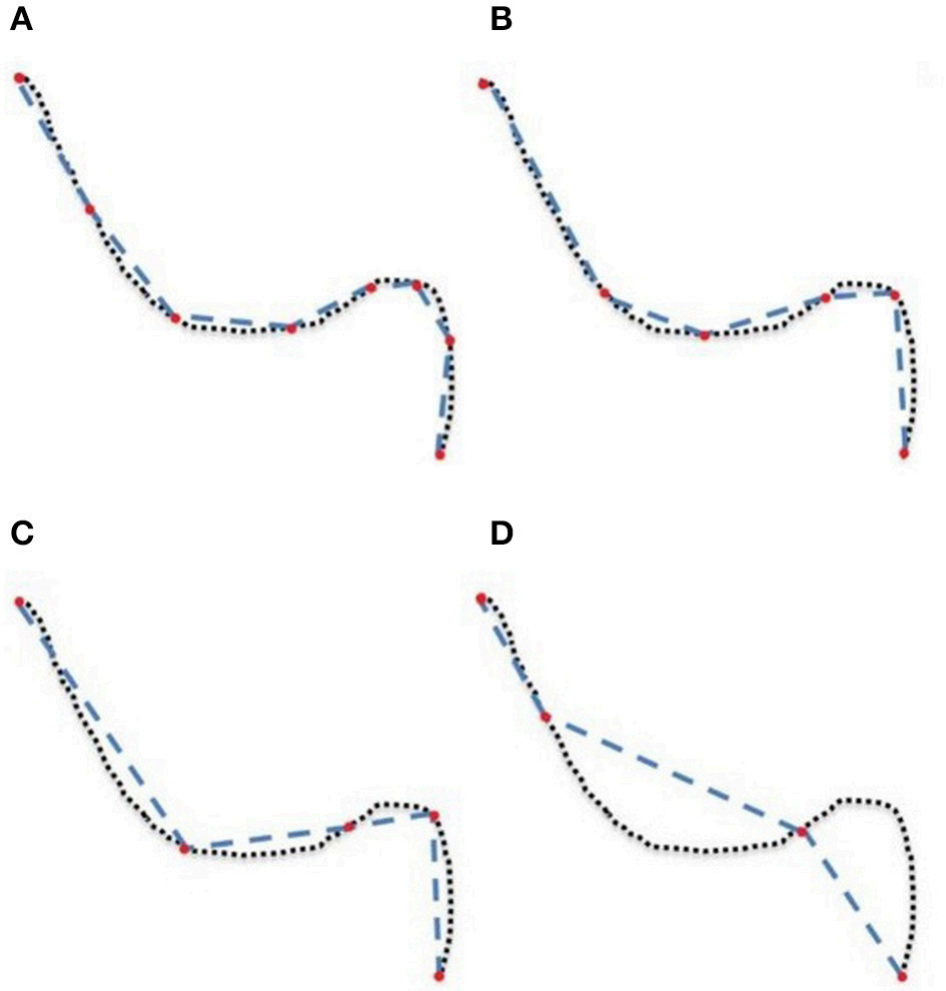
A simplified representation of a streamline containing 75 points (dotted line) linearized and now containing 8 **(A)**, 6 **(B)**, 5 **(C)**, and 4 **(D)** points (red), each forming an approximation of the original.

**Figure 2:**
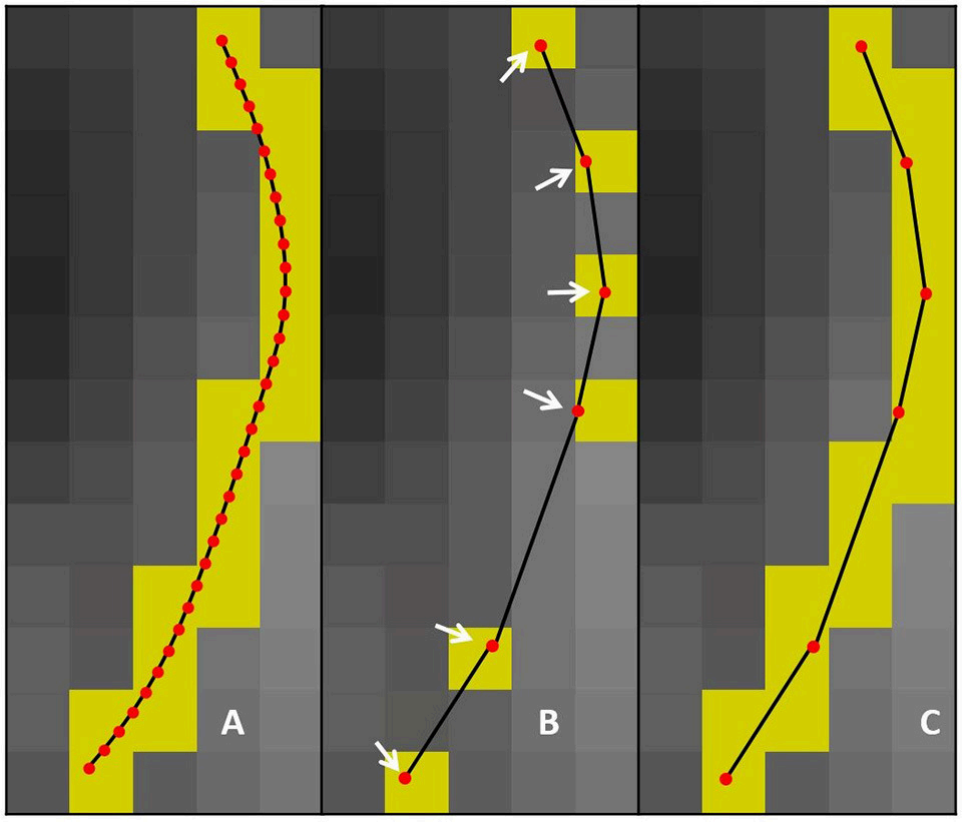
**(A)** uncompressed streamline: voxels found by the point-based technique are in yellow. **(B)** compressed streamline: arrows show points that were kept, voxels found by the point-based technique are in yellow. **(C)** compressed streamline: voxels found by the segment-based technique are in yellow.

The goal of this work is to integrate the linearization process presented in Presseau et al. (2015) into an existing application, and to provide methods that can correctly handle compressed streamlines. For example, interaction with streamlines and tractometry are the first two techniques that need to be adapted. In this work, “compression” only refers to the linearization presented by Presseau et al. (2015). That publication presented a more complex method to compress tractogram, the linearization was the step with the highest compression rate and was the fastest to execute. Some steps were only used when saving on disk and not available when the tractogram was loaded in RAM, so these steps were discarded. The linearization process consists of discarding collinear points using a maximum error threshold (MET), as shown in Figure 1. Once discarded, these points cannot be recovered. This mandates the use of adapted mechanisms to work with such files. As seen in Figure 2, all methods using the points of the streamline rather than segments will potentially be affected by streamlines with a non uniform or a large step size. It is also easy to imagine that tract-based statistics, such as mean FA along streamlines of a bundle, computed on such compressed representation will give biased statistics.

We successfully visualize 2M streamlines with a response rate between 15 and 25 frame per second. Then, we also show how to correctly filter and segment these compressed streamlines using regions of interest. Finally, we adapt tractometry methods to correctly handle linearized streamlines. In some cases, the proposed enhancements yield results that are even more robust than the classical point-based techniques.

## 2. Methods

### 2.1. MRI acquisition and experimental setup

Diffusion-weighted images (DWI) were acquired on a single volunteer along 64 uniformly distributed directions using a *b*-value of *b* = 1,000 s/mm^2^ and a single *b* = 0 s/mm^2^ image using the single-shot echo-planar imaging (EPI) sequence on a 1.5T SIEMENS Magnetom (128 × 128 matrix, 2 mm isotropic resolution, TR/TE 11,000/98 ms and GRAPPA factor 2). The study was performed according to the guidelines of the Internal Review Board of the Centre Hospitalier Universitaire de Sherbrooke (CHUS). Fractional Anisotropy (FA) was computed and the single fiber response function was estimated from all FA values above a 0.7 threshold within a white matter mask. The single fiber response was then used as an input for spherical deconvolution (Tournier et al., 2007; Descoteaux et al., 2009) to compute fiber ODFs of order 8. Using deterministic HARDI and streamline-based probabilistic HARDI tracking, 50 k, 100 k, 250 k, 500 k, 1 M, and 2 M streamlines were generated using a 0.5 mm step size and seeded in a WM mask computed by thresholding the FA at 0.1. For the experiments of Sections 2.2 and 2.3, tractograms were compressed using 5 different error thresholds, ranging from 0.1 to 0.5 mm.

For the experiments of Section 2.4, 27 bundles were extracted using the TractQuerier (Wassermann et al., 2016) from the 500 k streamlines tractogram. Segmentation is achieved using queries provided online (https://github.com/demianw/tract_querier) and ROIs defined by the Freesurfer cortical and white matter parcellation obtained from the T1-weighted image. This dataset is the same as the one used in publications of Presseau et al. (2015) and Houde et al. (2015). Each bundle was compressed using 9 different error thresholds (MET), ranging from 0.001 to 1 mm. Diffusion tensor-derived scalar measures, axial, radial, mean diffusivities (AD, RD, MD) and fractional anisotropy (FA), were computed using Dipy.

The load-time linearization algorithm was implemented in MI-Brain (http://www.imeka.ca/mi-brain), a MITK-based C++ software (Wolf et al., 2004) and tested on a desktop computer with an Intel 8 cores (i7) 3.20 GHz CPU, 16 GB of RAM and a NVIDIA GeForce GTX 295 GPU. The file format used was native MRtrix ^*^.tck for all the generated datasets.

### 2.2. Efficient loading and visualization

For efficient loading and visualization, the linearization of Presseau et al. (2015) was done at load-time. Once the streamlines are generated, collinear points become unnecessary and can be discarded for visualization purposes. Removing these points from the dataset reduces the file size, resulting in faster rendering of the tractogram without quality loss.

**Figure 3:**
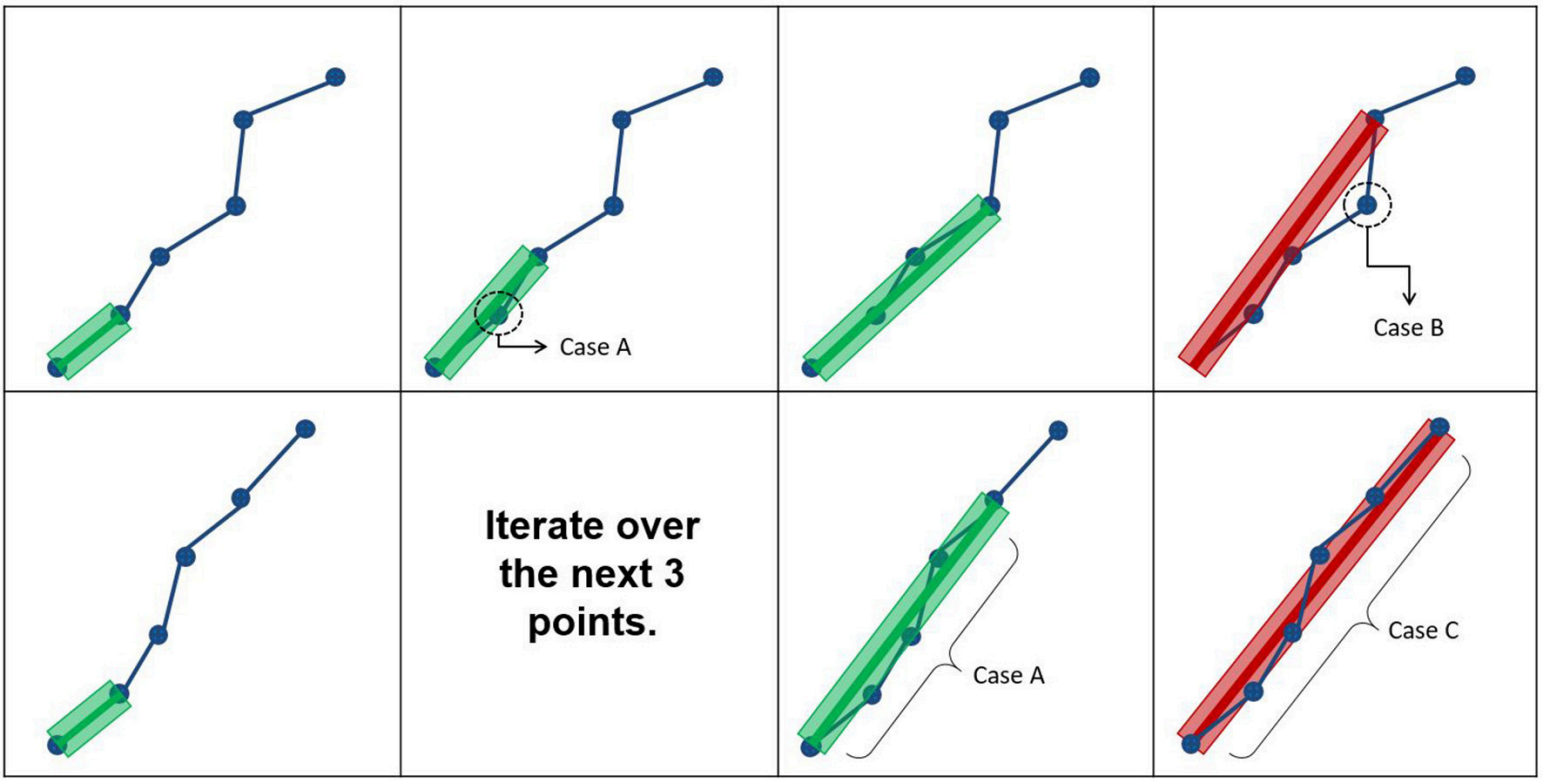
The first row represents how the maximum error threshold affects compression, while the second row represents how the maximum linearization distance affects compression. Case A occurs when all points are within the maximum error threshold and maximum linearization distance, meaning that the candidate point can be discarded. Case B arises when the current point breaks the maximum error threshold constraint, while respecting the maximum linearization distance. Case C is the opposite of Case B.

Perfectly collinear points are rare. This is why a maximum error threshold was used in the original method of Presseau et al. (2015). The value of the threshold is the main parameter of the linearization method. Based on Presseau et al. (2015) values between 0.1 and 0.5 mm were tested. The linearization process is shown in the first row of Figure 3. Simply put, to linearize a streamline, points are iteratively used to create a new, simpler line. At each step, all previous points (up to the previously kept point) are checked again (backward verification) to make sure that they are not farther from the line than the maximum error threshold. When a point cannot satisfy this constraint, it will be kept as a point on the compressed streamline, and the points between the previously kept point and the newly kept point are discarded. The process is then applied to all the remaining points, until the end of the streamline. The bulk of the processing time is spent in the backward verification phase.

In this work, an additional parameter is introduced to reduce the compression time and facilitate the adaptation of streamlines selection using ROIs. The maximum linearization distance (MLD) constraint limits the processing needed at load-time by avoiding long backward verifications. Applying this constraint helps reduce the number of operations at low cost. For the maximum linearization distance, 3 values were tested : 5, 10, and 25 mm. The behavior of these two parameters is represented in Figure 3 and the pseudocode is available in the Appendix (Supplementary Material).

There are 3 possible cases when iterating over the points of the streamline. Case IDs refer to Figure 3. Case A: all points between the current point and the previously kept point are within the maximum error threshold, and the current point respects the maximum linearization distance constraint. In this case, all points are mostly collinear, they do not add much information to the curve, and the distance from the previously kept point is not large enough to cause overhead. The linearization process will then proceed to analyze the next candidate point. Case B: a point in the backward verification process is outside of the maximum error threshold and the current point is within the maximum linearization distance. In this case, a point is breaking the collinearity constraint and, as such, adds information to the curve. Note that, in this case, the overhead of the backward verification is not problematic. Case C: all points currently checked are still within the maximum error threshold, but the current point is too far away from the previously kept point. In this case, all points are mostly collinear and don't add meaningful information to the curve, but an unconstrained linearization would start to cause overhead in the backward verification step. Cases B and C mean that linearization needs to be stopped for the current segment, and a new segment needs to be created. This linearization process source code is available in the free and open source project Dipy (http://dipy.org/) (Garyfallidis et al., 2014).

### 2.3. Interaction robust to compressed streamlines

**Figure 4:**
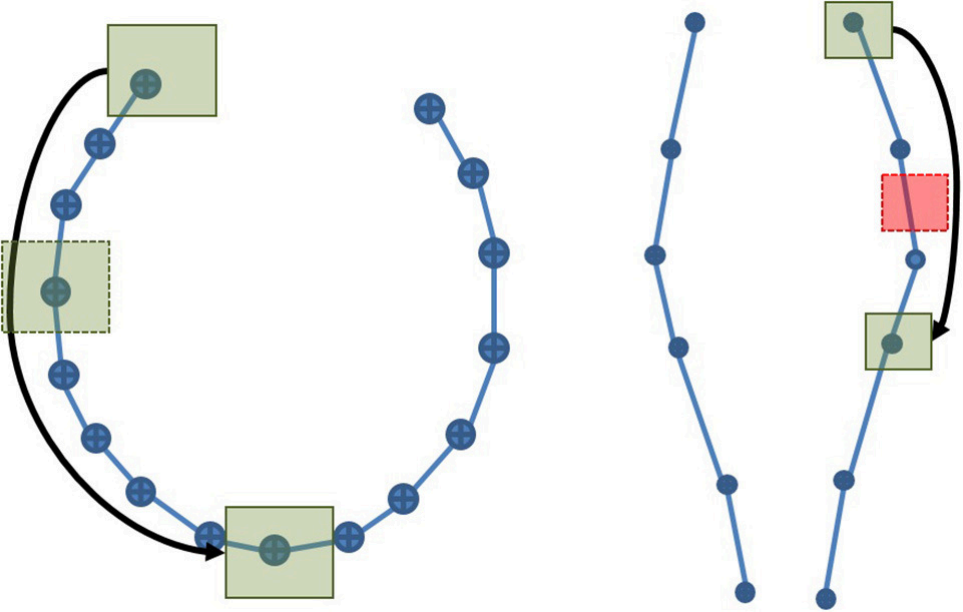
A simplified representation of the CC **(Left)** and the CST **(Right)**. The high curvature in the CC explains the low compression rate and thus the large number of points. The opposite phenomenon can be observed in the CST. If a ROI is placed along each streamline and the selection algorithm is point-based, the probability to obtain an inaccurate selection is higher in the CST than in the CC. As the maximum error threshold increases, the probability to miss streamlines increases in both, but remains higher in the CST.

Streamlines segmentation was done in MI-Brain on a 500 k streamlines tractogram, which was compressed using maximum error threshold values between 0.1 and 0.5 mm. Two cubic ROIs of 5 × 5 × 5 mm were used: one was placed in the region of the anterior posterior midbody of the corpus callosum (CC), while the other was placed in the cortico spinal tract (CST). The choice of these positions was done under the hypothesis that the compression ratio would vary according to curvature of the bundle. Since the CC is highly curved, it is less compressible, while the CST should be easily compressible, having a low curvature, as illustrated in Figure 4.

The term ROIs is used to define boxes and ellipsoids of any size and orientation or any complex surfaces in the graphical application. Our selection method works on any type of ROI since it is performed using polygon meshes that do not rely on a 3D grid. This is the basis of this type of method, which works in 3D space, trying to find at least one intersection with any type of geometrical object. This differs from the method presented in Section 2.4, which works in a grid and tries to identify all voxels touched by a streamline.

Using an approach based on points along with compressed streamlines will severely affect the results as a high number of points are missing from the dataset. The solution to this problem is to rely on the line segment between the points rather than the points themselves. By computing the intersection between the line segment and the ROIs (represented by polygon meshes in graphical application) the dependence on a small and uniform sampling along the streamlines is removed. However, this segment-based approach would necessitate a large amount of computing time as millions of streamlines contains tens of millions of line segments. To keep a responsive interaction, we use a progressive approach that reduces useless computation and still generates valid results. This approach is based on a coarse point-based filtering (as is done in most visualization tools), followed by a segment-based filtering to complete the set of selected streamlines.

This method, as implemented in MI-Brain, starts by finding all the points contained in the bounding box of the ROI using an octree, which is a fast spatial partitioning data structure (Samet and Webber, 1988). Each of the point in this subset is then tested to verify its inclusion in the ROI. These tests are based on VTK mesh and primitives tests and are not computationally demanding. This first part of the method yields the majority of the streamlines that intersect with the ROI, while being the fastest. The pseudocode is available in the Appendix (Supplementary Material).

The second phase of the progressive approach attempts to find streamlines that were missed by the point-based technique without going through all the segments in the dataset. Using the known maximum linearization distance described in Section 2.2, the ROI bounding box is extended by this maximum linearization distance into an extended neighborhood region. This extended neighborhood is guaranteed to contain at least one point of any segment intersecting the polygonal mesh, since segments cannot be longer than the maximum linearization distance. Points contained in the extended neighborhood are then extracted from the previously computed octree. Points belonging to already selected streamlines are not tested. The neighboring segments supported by the remaining points are then tested for intersection with the polygon mesh, using VTK functions.

**Figure 5:**
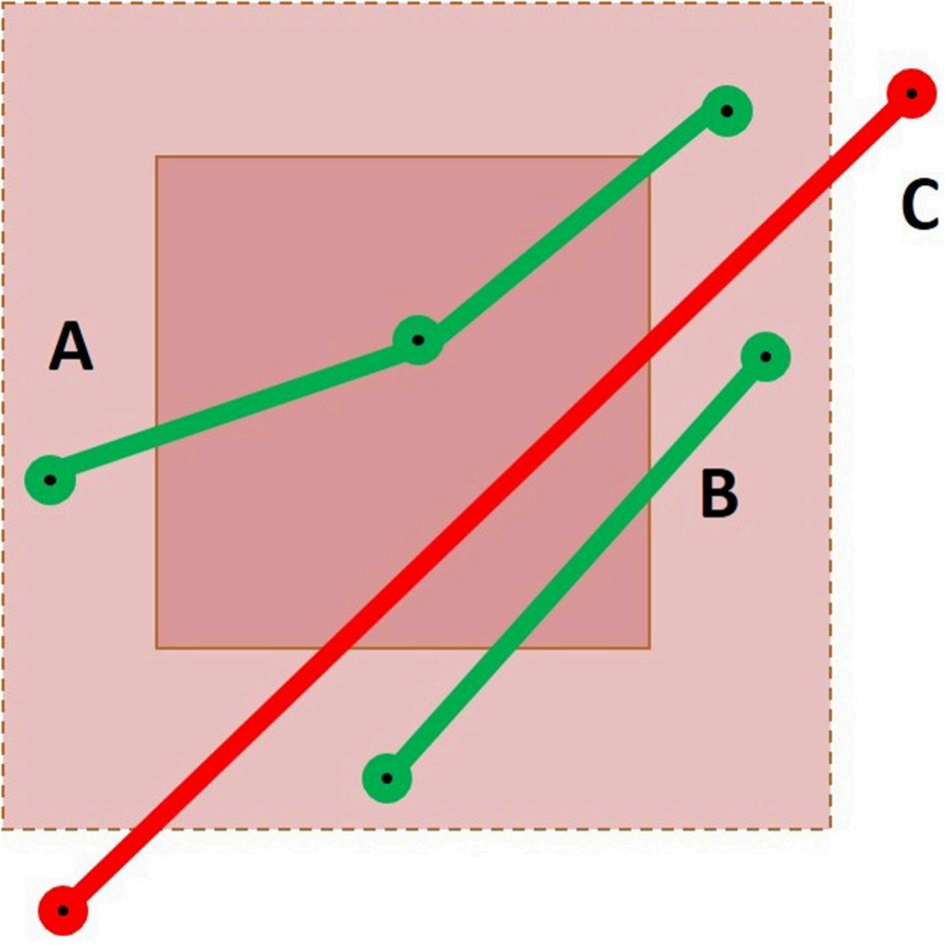
Representation of a cubic ROI and its extended neighborhood with three streamlines passing through it. Case A would result in a success for both the point-based and the segment-based methods. For Case B, only the segment-based method would succeed, using the mean segment length heuristic or using the maximal segment length calculation. Finally, Case C would only be selected by a segment-based method using the maximal segment length calculation.

To obtain all intersections correctly, the extended neighborhood size must be based on the maximal segment length in the dataset. The maximum linearization distance parameter introduced in Section 2.2 reduces the maximal size of the neighborhood. To accelerate the selection process, the size of the extended neighborhood can be based on the mean segment length of the dataset, but the results will not be complete. As demonstrated in Figure 5, three possible cases can occur. If the streamline has one point within the ROI itself, it will be selected correctly by the point-based method. If the streamline has no point within the ROI, but has points within the extended neighborhood, an intersection test will be performed to verify if the segment goes through the ROI. Finally, if the streamline has no point within both layers, the streamline will not be selected. The size of the extended neighborhood affects the computation time and the results of the selection. This is further discussed in the Appendix (Supplementary Material). If it is smaller than the longest segment in the tractogram, it is possible to miss streamlines.

### 2.4. Tractometry robust to compressed streamlines

As for the streamlines selection problem, when working with streamlines with non uniform step sizes, statistics and maps computed from such streamlines can be biased. This was clearly sketched in Figure 2. A classical way to compute the mean value of a diffusion-derived metric along a bundle of streamlines is to find out which voxels of the metric map are traversed by streamlines, and use this set of voxels as the support for the mean operation.

This operation is radically different from the one proposed in Section 2.3 since it is a rasterization operation on a uniform 3D grid as opposed to a complex polygon mesh. This rasterization operation aims to find all voxels traversed by streamlines, even when no point of the streamline is in a voxel and only its segment is crossing it Figure 2. Another notable difference is that Section 2.3 is about tractogram segmentation, while this section is about statistics of already segmented bundles, which contain a lot less streamlines than a whole brain tractogram, and usually does not have to be computed in real time in a graphical application. The pseudocode is available in the Appendix (Supplementary Material).

Of course, when this mapping from streamline to voxel is computed using only the points of the streamlines, techniques become susceptible to biases arising from varying step sizes. This will be referred as the voxel mapping bias. Varying step size can have many causes such as linearization (as presented earlier) or global tractography algorithms (Kreher et al., 2008; Fillard et al., 2009; Reisert et al., 2011; Lemkaddem et al., 2014).

To overcome the voxel mapping bias caused by variable step sizes, we replace the normal point-based technique by a Bresenham-style line integration technique (Bresenham, 1965; Amanatides and Woo, 1987). Instead of simply finding all voxels containing at least one point of the streamline, our technique computes the voxels intersected by each segment of the streamlines. All those voxels are then added to the tractometry computations, and the bias is therefore eliminated. This adapted method is also implemented in the free and open source project Dipy (http://dipy.org/) (Garyfallidis et al., 2014).

Using the dataset as described in Section 2.1 and both the basic, point-based technique and our proposed Bresenham-style technique, we calculated the mean values of diffusion metrics (AD, FA, MD, RD) for each bundle and error threshold combination. The basic point-based technique used the implementation available in Dipy (Garyfallidis et al., 2014). We also calculated the number of voxels occupied by those bundles, under each error threshold. Then, for each bundle and error threshold combination, we computed the difference between the uncompressed mean and the mean of each combination. This difference is then expressed as a percentage of the mean value over the uncompressed bundle.

## 3. Results

### 3.1. Efficient loading and visualization

**Figure 6:**
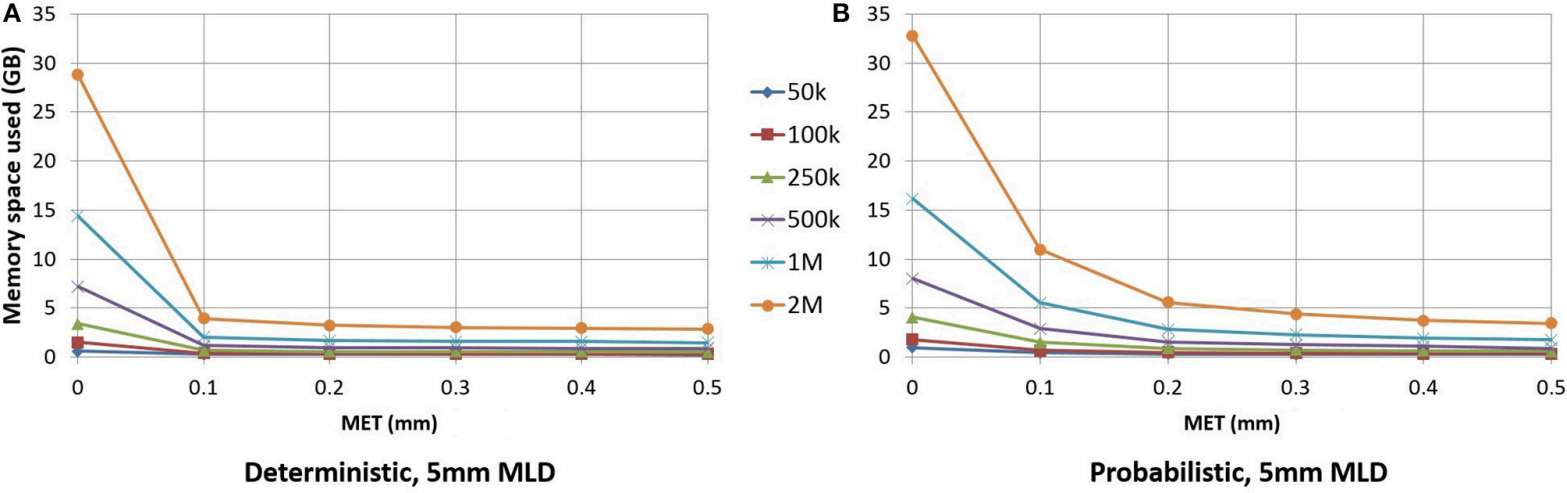
RAM space used by the application in relation to the maximum error threshold. Deterministic tractogram **(A)** and probabilistic tractogram **(B)** are shown and the Maximum Linearization Distance (MLD) is fixed at 5 mm for both types of tractogram.

As seen in Figure 6, for small maximum error threshold values, the linearization process discards up to 85% of the points for deterministic streamlines. The vast majority of points is removed with a 0.1 mm maximum error threshold, which is the lowest threshold we used for this part of the study. Compressing at higher thresholds, such as 0.4 or 0.5 mm, lead to insignificant gains in terms of disk or RAM space.

For an analysis offering a fair comparison to deterministic tracking, a 0.2 mm linearization threshold was employed for probabilistic streamlines. Figure 6 shows the differences between the deterministic and probabilistic streamlines; deterministic streamlines have a greater slope due to a lack of quick, local directional changes when tracking, thus allowing for better compression. Probabilistic streamlines have a much less smoother curve because probabilistic tracking makes sharper local changes in variable directions, leading to worse compression rates for a given maximum error threshold. The maximum linearization distance only having a small effect on compression rate, the same value can be used for deterministic and probabilistic tractograms. Descriptions of the optimal parameters for linearization are in the Appendix (Supplementary Material).

When linearization is unconstrained, an increase in maximum error threshold results in a quick increase in the linearization time. In this case, the maximum linearization distance parameter can constrain the process, controlling the linearization time, especially for high maximum error thresholds as shown in Table 1. The backward verification is the most time-consuming step in the algorithm. Therefore, by restricting the number of such verifications, numerous unnecessary operations are avoided, which accelerates the linearization step. Since the time difference between a constrained and an unconstrained linearization becomes large, the use of this constraint is necessary. A visual representation of the impact of constraining linearization is shown in Figure 9 in the Appendix (Supplementary Material).

**Table 1:** Comparaison of the compression time between an unconstrained linearization and a linearization constrained to 5 mm.

**MET (mm)**	**MLD = 5 mm (ms)**	**MLD = 10 mm (ms)**	**MLD = 25 mm (ms)**	**No constraint (ms)**
0.1	48,526	52,755	52,943	53,198
0.2	53,038	79,843	82,342	83,982
0.3	55,137	88,069	98,313	99,524
0.4	55,517	99,513	117,710	119,071
0.5	55,771	119,512	154,080	157,731

*The dataset used for the table was the tractogram with 2 millions deterministic streamlines*.

### 3.2. Interaction robust to compressed streamlines

Figure 4 showed the visual impact of compression on a schematic streamline, while also showing the importance of adapting the selection method to ensure that all streamlines are correctly selected and displayed. In neuroanatomical areas where fasciculi have a high curvature, e.g., the CC, a minimal 10–20% loss of selected streamlines was observed due to a less aggressive linearization in that bundle. The ability of the method to accurately select streamlines is influenced by both compression parameters, i.e., maximum error threshold and maximum linearization distance. Increasing one or the other increases the number of points being discarded, which in turn increases the number of streamlines missed by a purely point-based method. Tables 2, 3 demonstrate the effect of both compression parameters on the point-based selection method.

**Table 2:** Comparison of the number of streamlines selected in the CC, under various MET and MLD values.

**MET (mm)**	**MLD (mm)**	**Point-based (count)**	**Segment-based (Heuristic)**	**Segment-based (complete)**	**Missed streamlines (%)**
0	0	1,527	1,548	1,548	1.36
0.1	5	1,285	1,375	1,375	6.54
0.1	10	1,320	1,400	1,400	5.71
0.1	25	1,318	1,398	1,398	5.72
0.2	5	1,330	1,412	1,412	5.81
0.2	10	1,314	1,398	1,398	6.00
0.2	25	1,314	1,398	1,398	6.00
0.3	5	1,284	1,294	1,294	7.96
0.3	10	1,222	1,356	1,356	9.88
0.3	25	1,216	1,345	1,345	9.59

*“point-based” is the number of streamlines selected using the basic point-based technique, whereas “segment-based” represents the proposed technique. “Heuristic” means that the extended neighbor search was based on the mean segment length heuristic, while “Complete” implies searching in the maximal segment length neighborhood. “Missed streamlines” represents the percentage of streamlines found by the complete segment-based method but not by the point-based technique*.

**Table 3:** Comparison of the number of streamlines selected in the CST, under various MET and MLD values.

**MET (mm)**	**MLD (mm)**	**Point-based (count)**	**Segment-based (Heuristic)**	**Segment-based (complete)**	**Missed streamlines (%)**
0	0	1,605	1,655	1,655	3.02
0.1	5	1,076	1,562	1,562	31.11
0.1	10	885	1,467	1,478	40.12
0.1	25	885	1,460	1,471	39.83
0.2	5	1,075	1,536	1,536	30.01
0.2	10	791	1,431	1,441	45.10
0.2	25	788	1,390	1,404	43.87
0.3	5	977	1,504	1,504	35.03
0.3	10	627	1,334	1,350	53.55
0.3	25	618	1,316	1,332	53.60

*“point-based” is the number of streamlines selected using the basic point-based technique, whereas “segment-based” represents the proposed technique. “Heuristic” means that the extended neighbor search was based on the mean segment length heuristic, while “Complete” implies searching in the maximal segment length neighborhood. “Missed streamlines” represents the percentage of streamlines found by the complete segment-based method but not by the point-based technique*.

As both maximum error threshold (MET) and maximum linearization distance (MLD) increase, more streamlines are missed due to the increasing number of missing points. When no compression is done, using additional intersection tests only selects 3% more streamlines. However, even a maximum error threshold as low as 0.1 mm yields a loss of more than 30% of streamlines when the segment-based adaptation is not applied. The total number of selected streamlines varies as the parameters change. The small change in the streamline path caused by the compression explains that variation. Compression with a smaller maximum error threshold has less effect on the path but still modifies it slightly. Since it is fairly rare to linearize a long segment of a streamline, the heuristic is a good approximation of the complete calculation. Furthermore, the size of the box also affects these percentages, since a bigger ROI will increase the probability to encompass points, while a thin ROI would contain no point and only intersection tests would permit any selection.

**Figure 7:**
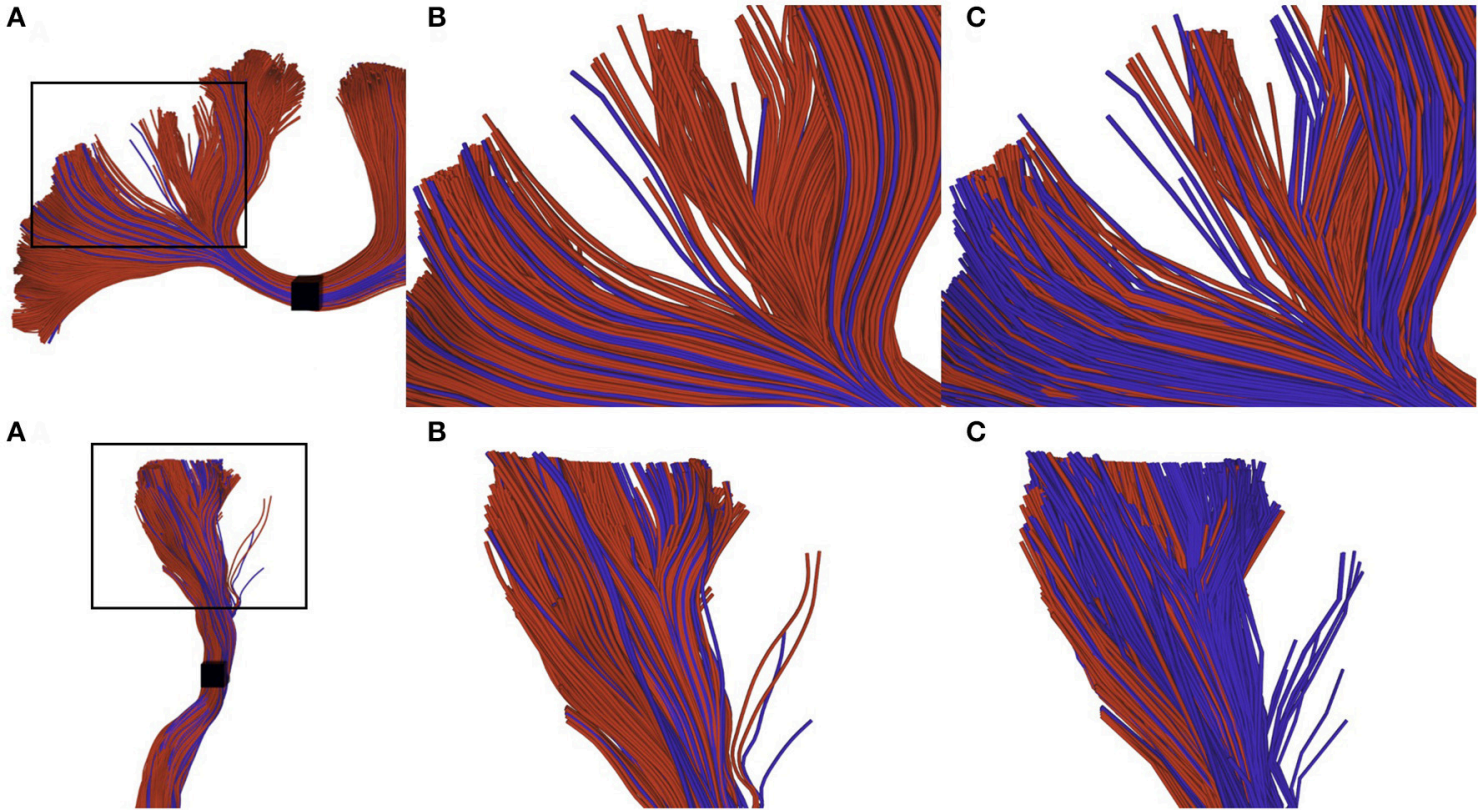
Visual impact of compression on selected streamlines. The first row represents a small part of a corpus callosum (region of the anterior posterior midbody), while the second row represents a segmented cortico spinal tract. **(A)** zoomed subsection of the bundles that we want to observe. **(B)** selection of uncompressed streamlines: red streamlines are selected using a point-based method, blue streamlines are those that can only be selected by a segment-based method. **(C)** selection of compressed streamlines, color scheme is the same as in **(B)**. We can observe that the proportion of blue streamlines is higher, since the point-based method has a higher probability to fail when processing compressed streamlines.

The difference of selection results between techniques is more difficult to observe on a real tractogram, since they normally use a small maximum error threshold and are denser. Figure 7 shows the importance of the adapted selection method, since the only visual effect is in the streamline density. Using a point-based method (red streamlines), a significant number of streamlines can be missed, leading to an erroneous streamlines count. The blue streamlines were selected using our segment-based approach. Note that count is only used as a qualitative measure of the effectiveness of the selection method.

### 3.3. Tractometry robust to compressed streamlines

Table 4 shows the mean FA values for the left CST bundle, for all error thresholds tested. From this table, it is clear that the basic point-based technique is biased, with higher error thresholds having significant differences between both techniques. For example, the difference between the value for the uncompressed bundle and the value when compressed with a 1mm maximum error threshold is 0.08062 (14.5% of the original value) for the point-based technique. For the segment-based technique, this difference is 0.00168, representing 0.3% of the original value.

**Table 4:** Mean FA values computed over the left CST, for various error thresholds.

	**Original**	**0.001 mm**	**0.005 mm**	**0.01 mm**	**0.02 mm**	**0.05 mm**	**0.1 mm**	**0.2 mm**	**0.5 mm**	**1 mm**
Points	0.55098	0.55006	0.54011	0.53195	0.52529	0.51808	0.51221	0.50902	0.49249	0.47406
Segments	0.55113	0.55113	0.55113	0.55117	0.55126	0.55144	0.55182	0.55265	0.55366	0.55281

*Original represents the uncompressed bundle. The “Points” row shows the results for the classical, point-based technique, whereas the “Segments” line shows the results for our proposed technique. Note that the point-based results are more variable, and therefore, less reliable. The precision of the results was chosen to illustrate the magnitude of changes across error thresholds (MET) tested*.

The reader might also have observed that the point-based and segment-based techniques do not provide the same values for the original, uncompressed bundle. This is due to the segment-based technique finding all voxels intersected by a streamline, even when no point of the streamline resides in the voxel. For example, a streamline might simply go over the corner of a voxel without actually having a point in that voxel, see Figure 2. Since the segment-based technique finds all such voxels and includes them in computations, there is a slight difference in the resulting uncompressed bundle mean values.

Another trend is the steady drop in the mean FA value measured with the point-based technique when increasing the compression error threshold. The main cause of this phenomenon is that regions in straight parts of the bundle lose more points when compared to curved regions. In the CST for example, the mean FA value in straight sections of a bundle tend to be denser and only oriented in one direction, which increases the FA for those voxels. Meanwhile, curved sections tend to be near crossing with other bundles, which might reduce the FA in those regions.

Normally, the average value of a metric should take into account all voxels that are traversed by at least one streamline. This would generate a representative average value. This average can either be based on a binarized map (i.e., each traversed voxel is used once), or weighted by using the number of times each voxel is traversed. Depending on the use case, this weighting generates better averages, since regions that are more densely packed will have more influence on the average value (Yeatman et al., 2012). However, once streamlines are linearized, the discarded points in the straighter parts are not taken into account in the computed average, resulting in a disparity between the computed average and the actual average. This disparity is increased in the case of a weighted average, since straighter parts are normally more packed. Using a segment-based approach will prevent such disparity, since the discarded points are no longer needed to obtain the true voxel importance in the computed average.

**Figure 8:**
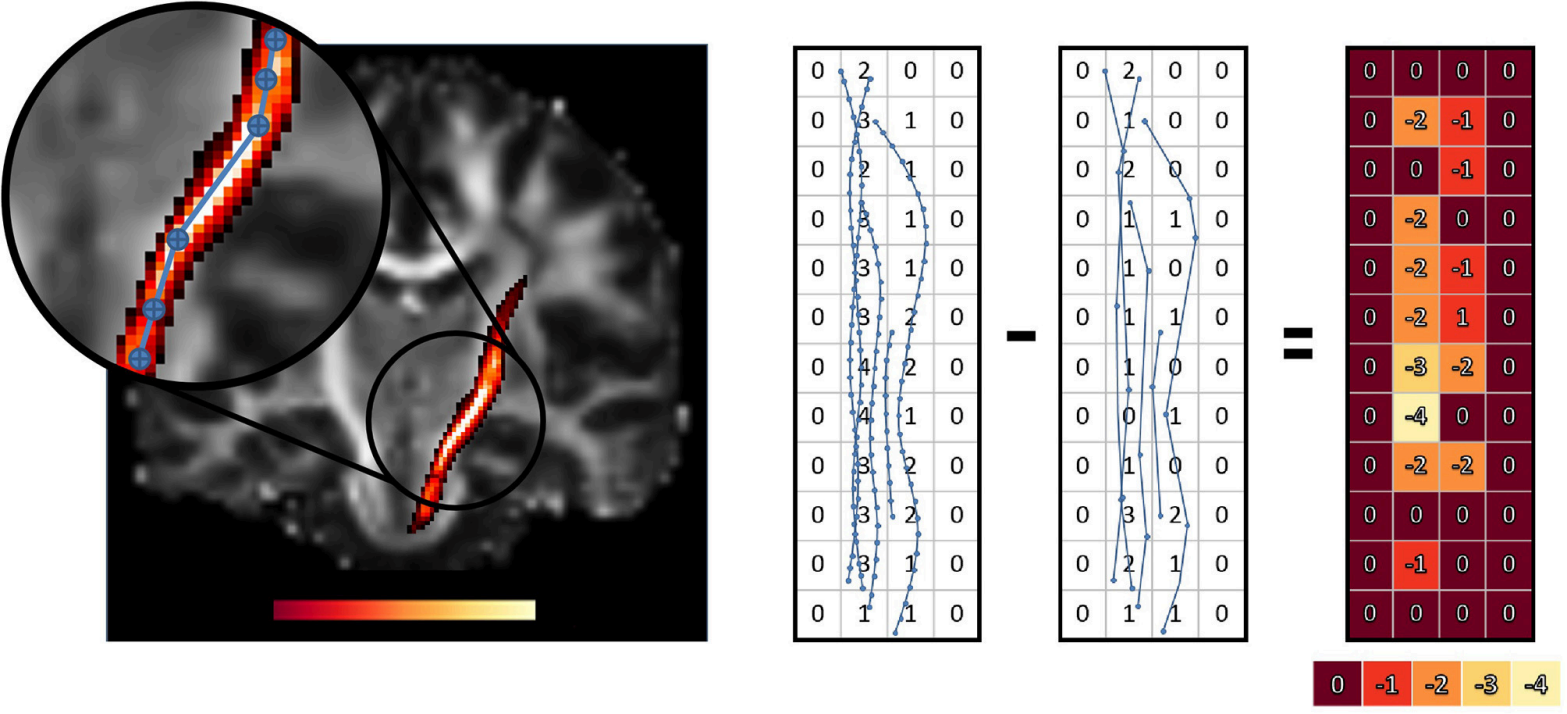
Difference in voxel weighting between the original and compressed left CST bundle, using the point-based technique. Whiter regions have a higher weight loss and also highlight the nonuniformity of the weight loss for the statistic of the bundles. The zoomed portion shows how curvature affect compression which directly influence the weight loss. The simplified view on the right helps to understand how a point-based method reduces streamlines density in some voxels when we compress streamlines. The overall influence of these voxels on the weighted average will decrease.

This can be seen in Figure 8. White colored regions lose the most weight in the average. This means that voxels in whiter regions will have less importance on the computed averages than what they should have had. This impact will vary for each bundle, since it depends on the shape of the bundle, which will impact tractometry.

To verify that the segment-based technique works correctly for various shapes and sizes of bundles, we define ${\overset{-}{M}}_{b}^{c}$ as the mean value of metric *M* over bundle *b* for compression threshold *c*, with *M* ∈ {AD, FA, MD, RD, Volume}. *b* is one of the 27 bundles produced by TractQuerier (Wassermann et al., 2016) and *c* represents one of the 9 error thresholds. *c* can also take the value of 0, which represents the uncompressed bundle. Then, $R\left({\overset{-}{M}}_{b}^{c}\right)=\frac{\left|{\overset{-}{M}}_{b}^{c}-{\overset{-}{M}}_{b}^{0}\right|}{\left|{\overset{-}{M}}_{b}^{0}\right|}$ represents how much ${\overset{-}{M}}_{b}^{c}$ differs from ${\overset{-}{M}}_{b}^{0}$ with regard to the original value of ${\overset{-}{M}}_{b}^{0}$.

Table 5 shows the mean and standard deviation of $R\left({\overset{-}{M}}_{b}^{c}\right)$ (difference between the original mean value of the metric and the mean value after compression) for all bundles *b* and each value *c*. From Table 5, we see that the segment-based technique is always more robust and less variable than the point-based technique. For very small error thresholds (0.001 mm, 0.01 mm), the mean and standard deviation of the segment-based technique are always at least 2 orders of magnitude smaller than the one of the point-based technique. For a reasonable error threshold (0.1 mm), segment-based is always 2 to 3 times more robust. For a large error threshold (1 mm), it depends on the metric under consideration. Generally, the segment-based technique is 1.5–2 times more robust.

**Table 5:** Mean and standard deviation of $R\left({\overset{-}{M}}_{b}^{c}\right)$ for the point-based (pts) and segment-based (seg) techniques.

		**0.001 mm**	**0.01 mm**	**0.1 mm**	**1 mm**
AD	pts	(3.5 ± 4.4) × 10^−4^	(8.4 ± 6.5) × 10^−3^	(1.6 ± 1.2) × 10^−2^	(2.3 ± 1.5) × 10^−3^
	seg	(4.4 ± 5.1) × 10^−7^	(5.3 ± 5.4) × 10^−5^	(8.9 ± 9.9) × 10^−4^	(1.2 ± 1.2) × 10^−3^
FA	pts	(0.0 ± 1.1) × 10^−3^	(2.2 ± 1.5) × 10^−2^	(6.1 ± 2.5) × 10^−2^	(1.2 ± 0.31) × 10^−1^
	seg	(1.1 ± 1.8) × 10^−6^	(7.0 ± 5.8) × 10^−6^	(1.4 ± 1.1) × 10^−3^	(2.2 ± 1.5) × 10^−2^
MD	pts	(1.4 ± 1.6) × 10^−4^	(2.8 ± 2.8) × 10^−3^	(1.3 ± 0.7) × 10^−2^	(4.7 ± 2.4) × 10^−2^
	seg	(5.4 ± 9.3) × 10^−7^	(5.5 ± 8.8) × 10^−5^	(0.9 ± 1.4) × 10^−3^	(0.8 ± 1.4) × 10^−3^
RD	pts	(5.3 ± 6.7) × 10^−4^	(1.2 ± 1.0) × 10^−2^	(4.1 ± 1.8) × 10^−5^	(1.0 ± 0.4) × 10^−1^
	seg	(0.9 ± 1.6) × 10^−6^	(0.8 ± 1.4) × 10^−4^	(1.2 ± 2.2) × 10^−3^	(1.4 ± 2.1) × 10^−2^
Vol	pts	(4.0 ± 1.0) × 10^−3^	(8.5 ± 2.0) × 10^−2^	(4.1 ± 0.7) × 10^−1^	(7.1 ± 0.6) × 10^−1^
	seg	(0.0 ± 0.0) × 10^0^	(3.6 ± 3.2) × 10^−4^	(1.3 ± 0.9) × 10^−3^	(5.0 ± 1.7) × 10^−2^

*Each column presents a specific error threshold *c*, and each line represents a metric *M*. Some intermediate error thresholds were omitted to simplify the table. Significant numbers are kept according to the magnitude of the standard deviation*.

## 4. Discussion

### 4.1. Summary of the contributions

#### 4.1.1. Efficient loading and visualization

Compressing streamlines datasets can ease the transmission of such datasets over networks and can make them more easily loadable in a visualization application. One of our contribution is the implementation of the linearization phase directly in the loading code of a visualization application (MI-Brain). This contribution removes the need to compress datasets before loading them, while keeping the benefits in interaction performance having lighter datasets. When implemented in such a way, the linearization phase increases loading time, but reduces subsequent parts of the pipeline, such as initialization of the data structures and data transfers to the GPU, resulting in the reduction of the overall time before display. Another contribution is the introduction of the maximum linearization distance parameters constraint the time needed for linearization by limiting the number of backward verification. The linearization method is also available in Dipy (dipy.tracking.streamlinespeed).

The number of points removed from the tractogram does not impact the visualization and enables large datasets to be visualized on medium-range computers. Compression of streamlines generates new questions such as the minimal level of details needed to perform virtual manual dissection or the accuracy of the path recovered during tracking vs. the linearize version. These questions are very similar to issues in image or video compression used for scientific purposes and depending on the use or the research, the answers vary greatly. Different needs will lead to different parameters for tractogram compression, or the absence of compression. More detailed descriptions of the optimal parameters for linearization and their consequences are in the Appendix (Supplementary Material).

#### 4.1.2. Interaction robust to compressed tractograms

The removal of points on streamlines impacts the subsequent segmentation when the underlying technique is point-based. The proposed segment-based approach solves this issue without affecting the interaction speed. Using the segment-based method can even improve the selection of uncompressed streamlines, since the step size is never infinitely small and some streamlines can still be missed using the point-based technique. The use of this approach is necessary to ensure that streamlines compression will not impact manual segmentation or any further processing based on streamlines.

When performing a virtual manual dissection with diffusion tractography, researchers tend to use tractogram with millions of streamlines to ensure a proper recovery of the desired bundle shape and spatial coverage. Compression is a good approach to enable researchers to use tractogram with more streamlines, developing methods robust to non uniform step size or compressed tractogram is crucial to avoid biased results. One of our contribution is the implementation of the robust interaction method in the visualization application MI-Brain.

It is important to mention that linearization does not have a uniform impact across the whole tractogram, because the curvature of each streamline will affect the amount of points removed. For example, research that perform analysis on multiple bundles could create a bias in their segmentation that vary between each bundle. More informations about the effect of linearization on the interaction with tractograms is available in the Appendix (Supplementary Material).

#### 4.1.3. Tractometry robust to compressed bundles

Adapting tractometry is also necessary to correctly handle compressed streamlines datasets. Even in the case of uncompressed streamlines, it was shown that segment-based tractometry was more reliable, since it could identify small voxels hosting a short part of a streamiine, even if no point of that streamline was present in said voxel. It may be argued that including such “unimportant voxels” could introduce some other kind of bias in the tractometry results. From preliminary tests, such a bias introduces almost no variability in the final metrics.

The robustness of tractometry metrics is important to ensure that derived statistics are not biased. Biased statistics could have adverse impacts on further analyses, which could for example generate false observations when studying neurogenerative diseases. Furthermore, advanced tractometry techniques, such as Yeatman et al. (2012) and Cousineau et al. (2016), now compute multiple metrics values along a bundle of streamlines, instead of computing a single value over the whole bundle. In this case, having a non-uniform importance for each section of the bundle could lead to varying errors along the bundles, which could also lead to erroneous conclusions. One of our contribution is the implementation of the robust tractometry method in the Dipy library (dipy.tracking.vox2track) to help the community to reduce potential biases in future analysis using tractometry.

#### 4.1.4. File format for compressed tractography datasets

Since compression modifies the streamlines, and most tools are not adapted to this, a dedicated format, or at least a dedicated tag in an existing format is needed to store compressed streamlines datasets. The existing format from TrackVis (trk file) could support such tag. This would prevent potentially invisible errors when using unadapted tractometry or bundle dissection tools with compressed streamlines. By having a dedicated format, tools developers would need to add support, and would therefore be made aware of the pitfalls of compressed streamlines. Using compressed data with unadapted pipelines can cause biased results because such a high proportion of points were discarded from the tractogram. Such results could lead to erroneous conclusions when compared to conclusions arising from a pipeline taking the compression into account.

Finally, a new specific file format could prevent some confusion about the size of a dataset. Currently, most people know the approximate size of a tractogram if they know the number of streamlines and the step size. With a streamlines-specific compression algorithm, this could completely change how people judge a file size. Depending on the compression parameters, a file can use between 8 and 15 times less disk space than usual. For instance, when tractograms are shared between labs or for online databases, a format for compressed datasets would help. Not having such a dedicated file format could otherwise lead one to think the file is broken or sparse.

The way data is structured in a tractogram makes it hard to reduce its size in an efficient way. In a tractogram file (^*^.trk, ^*^.tck, ^*^.vtk) each streamlines is independent and stored separately in a random order. Therefore, a lot of methods commonly used for meshes cannot be applied to streamlines. Other compression methods to compress even more the data on disk have been explored in Presseau et al. (2015), but these methods are time consuming and provide little advantages over linearization. Since the goal of this article was to deal with visualization and tractometry after the streamlines have been compressed, which mean the data need to be loaded in memory or even send to the GPU, the potential compression methods for data saved on disk were not discussed.

## 5. Conclusion

The advantages of tractography datasets compression for interactive exploration or data storage are compelling. However, current point-based methods are not able to robustly handle such files. Algorithmic segment-based adaptations are crucial before their use can become widespread. It is critically important to adapt streamlines segmentation and tractometry techniques to enable them to correctly handle compressed streamlines and non uniform sampling of points along streamlines. Without this adaptation, all techniques that rely on a point-based analysis of the streamlines will yield biased, if not simply incorrect results. Since the size of streamlines datasets is expected to continue increasing in the coming years, we expect the use of compressed streamlines to gain more traction. We also expect that more tractography techniques will be able to generate streamlines with variable step sizes (Lemkaddem et al., 2014; Schreiber et al., 2014), to account for various factors and perform global tractography-like algorithms based on a lower number of points. In general, major software tools will have to follow and adapt their techniques to ensure that analysis ran on those files will still be valid and accurately reflect the underlying streamline structure. A dedicated file format to contain compressed streamlines or streamlines with an irregular step size would prevent many potential problems.

Using all techniques presented in this paper can improve the experience of the user with faster and easier interaction. Furthermore, the disk space saved using compressed streamlines is important. For example, 100 tractograms, each having 500 k streamlines, can now fit on an 8 GB USB stick instead of needing more than 80 GB of space. With these adaptations, millions of streamlines can be loaded and interacted with on a medium-range laptop, making 3D streamlines visualization, segmentation and tractometry available to a wider audience. The implementation in the graphical application MI-Brain or in the free open-source library Dipy is a contribution aiming to put forward theses improvements.

## Author contributions

Study conception and design: MD, FR, and JH; acquisition of data and critical revision: MD and JH; analysis and interpretation of data and drafting of manuscript: FR and JH.

### Conflict of interest statement

The authors declare that the research was conducted in the absence of any commercial or financial relationships that could be construed as a potential conflict of interest.
