# Supplementary material for: Visualization, Interaction and Tractometry: Dealing with Millions of Streamlines from Diffusion MRI Tractography
Source: Front Neuroinform. 2017 Jun 26;11:42. doi: 10.3389/fninf.2017.00042 (PMC5483435; doi:10.3389/fninf.2017.00042)
Supplement: Supplementary file 1 [file DataSheet1.pdf]

6 APPENDIX

6.1 OPTIMAL PARAMETERS FOR EFFICIENT LINEARIZATION

Maximum error threshold Using a 0.1mm threshold for the linearization phase removes the majority of points of a deterministic tractogram. Larger thresholds could cause voxel shifts and slightly modify the actual path of a streamline. This unwanted effect can be observed, but the consequences are limited even at high error thresholds such as 0.5mm. Using a low threshold incurs a limited compression time while still removing most points. Overall, the load-time linearization of a tractogram reduces the waiting period before visualization and interaction. The initial drop in Figure 9 shows that compression, even, as a supplementary step, globally reduces the waiting time before visualization or interaction.

To have comparable results in terms of compression ratio and compression time, the optimal maximum error threshold for probabilistic streamlines is slightly higher. Using a 0.1mm threshold, only 65% of the points of a probabilistic tractogram were discarded. This is caused by the frequent local direction changes inherent to probabilistic tracking, causing the linearization process to fail to remove lots of consecutive points. The optimal threshold for this type of tractogram was found to be 0.2mm. A higher threshold would encounter the same issues as high thresholds with deterministic streamlines, such as a longer compression time, potential visual differences, and very small gains in term of disk or RAM space.

During our experimentation only a human brain was used and the value for our optimal maximum error threshold value is based on the average size and shape of human tractogram. In a situation where a tractogram is much smaller (infants or small animals for example) the choice of parameter should be made based on its dimensions and resolution. We suggest that the maximum error threshold should be between one tenth and one twentieth of the resolution of the volume in which the tracking was computed.

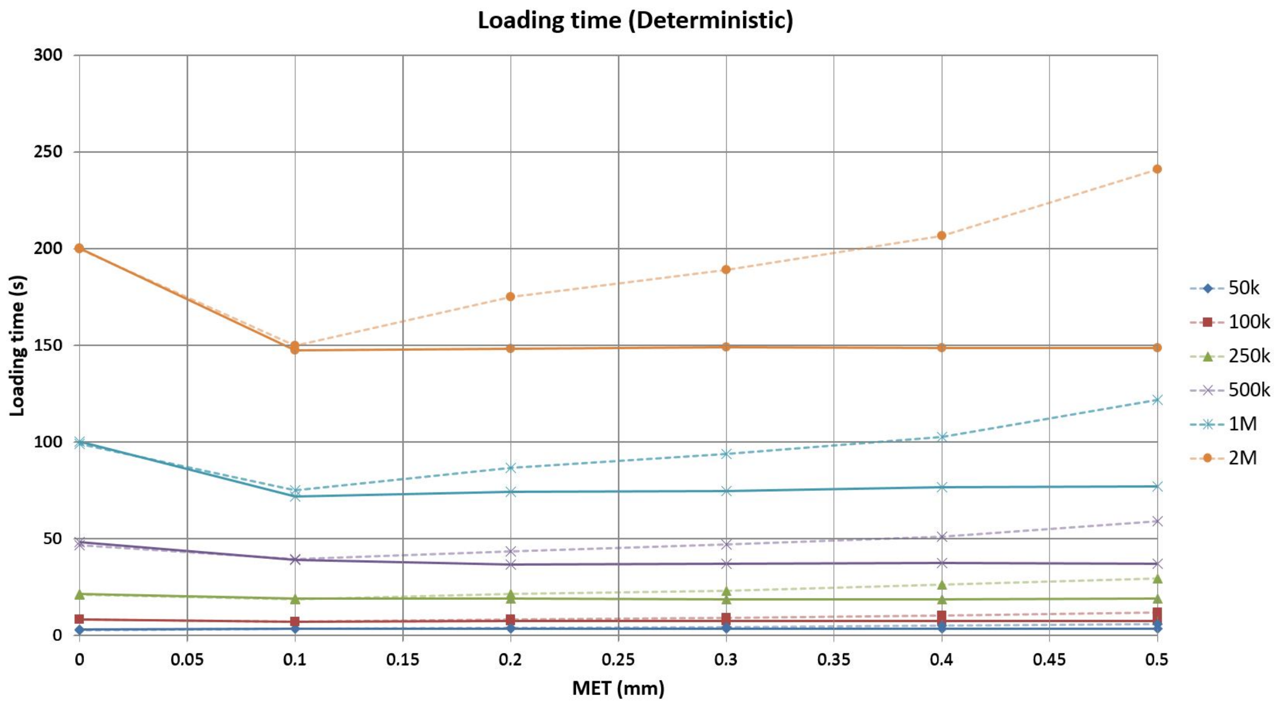

Figure 9. Loading time, with the maximum linearization distance fixed at 5mm, in function of the error threshold. The dashed line represents the loading time without the maximum linearization distance constraint. All the tractograms are deterministic.

Maximum linearization distance An optimal choice for the maximum linearization distance was found to be 5mm, both for deterministic and probabilistic datasets. Since most iterative tracking algorithms use a step size under 1mm, a 5mm maximum linearization distance is often enough to remove an important number of points. As the step size gets smaller, the necessity for the maximum linearization distance parameter becomes greater to prevent endless compression time. The linearization being done at load-time in a visualization software, it is important to limit its duration so that user experience is not degraded

Using such a value (5mm) for the maximum linearization distance leads to a smaller probability of missing streamlines when using the mean segment length based heuristic for the neighborhood exploration as demonstrated in Table 2 and 3. Adding intersections verifications for segments within a small extended neighborhood (heuristic) is enough to select 95% of the streamlines correctly and 100% if the complete extended neighborhood is used. Since the complete ROI intersection test uses the maximum segment length in the whole tractogram, constraining the maximum segment length to 5mm with the maximum linearization distance parameter reduces the potential number of segments to test for intersection.

Figure 10 demonstrates how both neighborhood definitions affect selection time and shows how the heuristic drastically reduces the computation time for streamlines selection, by limiting the number of intersection tests to perform. The first two bars of each group represent the selection time using the mean segment length search, and the two last represent the selection using the maximum segment length search. At low maximum error thresholds or low maximum linearization distances, the time is relatively short. As either constraint increases, selection time using the maximum segment length search increases drastically.

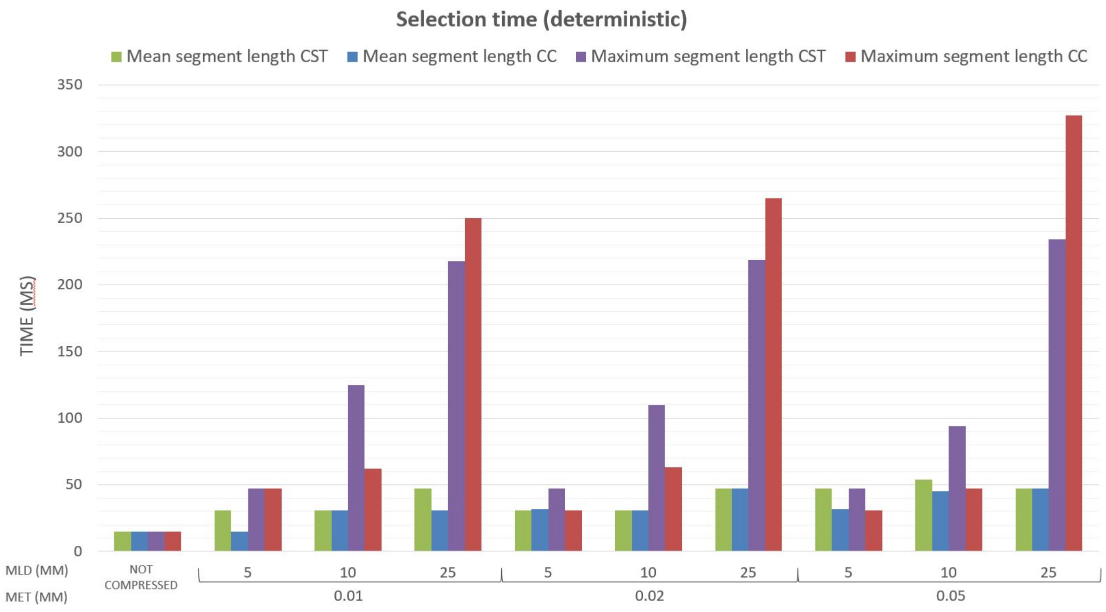

Figure 10. Selection time depending of the heuristic chosen to define the neighborhood used for intersection tests. Results are shown for deterministic streamlines.

The heuristic can be used as an approximation during interaction, while the complete calculation can be used only to visualize or obtain final results. At higher compression rates, the approximation using the mean segment length is the only way to achieve real-time selection with compressed streamlines while maintaining mostly accurate results. This time factor was important to take into consideration when choosing optimal compression parameters. In a situation where selection needs to be fully accurate and time is less of a concern, the maximum segment length should always be used instead of the heuristic.

As mentioned previously, the experiments presented in this paper were done using human datasets. Every parameters expressed in millimeters should be adapted if a different type of data is used. We suggest that the maximum linearization distance should be between one tenth and one twentieth of the total dimensions in mm of the volume in which the tracking was computed.

6.2 PSEUDOCODE FOR LINEARIZATION

```
Inputs:
  1. P, sequence of 3D points (one streamline)
  2. Maximum error threshold (MET), float
  3. Maximum linearization distance (MLD), float
Result: compressStreamlines, sequence of 3D points (one streamline)

Function Linearization(P, MET, MLD):
    i = 0, j = 1, n = nbr points in P
    ADD P_i to compressStreamlines
    while i < n do
        if distance between P_i and P_{i+j} > MLD then
            ADD P_{i+j-1} to compressStreamlines
            i = i+j-1
            j = 1
            CONTINUE
        end
        if i+j == n then
            ADD P_{i+j} to compressStreamlines
            i = i+j
            BREAK
        end
        for k between i and i+j do
            if distance between line(P_i, P_{i+j}) and P_k > MET then
                ADD P_{i+j-1}
                i = i+j-1
                j = 1
                BREAK
            else
                CONTINUE
            end
            j += 1
        end
    end
    return compressStreamlines
```

Algorithm 1: Simplified linearization algorithm

6.3 PSEUDOCODE FOR INTERACTION

```
Inputs:
  1. T, sequence of 3D lines (one tractogram)
  2. Maximum linearization distance (MLD), float
  3. ROI, a complex 3D polygon mesh
Result: selectedStreamlines, sequence of streamlines

Function SelectStreamlinesInROI(T, MLD, ROI):
    ROIBounds = ComputeBounds(ROI, 0)
    ROIExtendedBounds = ComputeBounds(ROI, MLD/2)
    pointsInBounds = GetPointsInBounds(T, ROIBounds)
    pointsInExtendedBounds = GetPointsInBounds(T, ROIExtendedBounds)
    for each P in pointsInBounds do
        if P is within ROI then
            ADD AssociatedStreamlines(P) to selectedStreamlines
        end
    end

    for each P in pointsInExtendedBounds do
        if IntersectWithROI(P, T, ROI then
            ADD AssociatedStreamlines(P) to selectedStreamlines
        end
    end
    return selectedStreamlines

Function ComputeBounds(ROI, distance):
    min_xyz = min_x(ROI) - distance, min_y(ROI) - distance, min_z(ROI) - distance
    max_xyz = max_x(ROI) + distance, max_y(ROI) + distance, max_z(ROI) + distance
    return min_xyz, max_xyz

Function GetPointsInBounds(T, bounds):
    octree = CreateOctree(T)
    listOfPoints = octree.getPoints(bounds)
    return listOfPoints

Function IntersectWithROI(P_i, T, ROI):
    P_{i+1} = getNextPointOnStreamlines(T, P_i)
    P_{i-1} = getPreviousPointOnStreamlines(T, P_i)
    for every polygon in ROI do
        if line(P_{i+1}, P_i) or line(P_{i-1}, P_i) intersect current polygon then
            return True
        end
    end
    return False
```

Algorithm 2: Simplified linearization algorithm

6.4 PSEUDOCODE FOR BRESENHAM-STYLE LINE INTEGRATION

```
Inputs: P, sequence of 3D points (one streamline)
Result: traversedVoxels, list of voxel indices

Function LineIntegration(P):
    ADD voxel(P_0) to traversedVoxels
    n = nbr points in P
    for i in [0..n-2] do
        if voxel(P_i) == voxel(P_{i+1}) then
            Continue to next pair of points
        end
        direction = P_{i+1} − P_i
        currentPoint = P_i
        remainingDistance = Norm(direction)
        while True do
            nextEdge = GetNextEdge(currentPoint, direction)
            lengthRatio = abs((nextEdge - currentPoint) / direction)
            smallestLengthRatio = MinimalComponent(lengthRatio)
            remainingDistance -= smallestLengthRatio * Norm(direction)
            if full length of segment was explored then
                BREAK
            end
            currentPoint += (smallestLengthRatio + ϵ) * direction
            ADD Voxel(currentPoint) to traversedVoxels
        end
    end
    return traversedVoxels

Function GetNextEdge(inputPoint, direction):
    nextEdge = (0, 0, 0)
    for dim in [0..2] do
        if direction[dim] ≥ 0 then
            nextEdge[dim] = ceil(inputPoint[dim])
        else
            nextEdge[dim] = floor(inputPoint[dim])
        end
    end
    return nextEdge
```

Algorithm 3: Simplified line integration algorithm
